# Emotional Responses to Music: Shifts in Frontal Brain Asymmetry Mark Periods of Musical Change

**DOI:** 10.3389/fpsyg.2017.02044

**Published:** 2017-12-04

**Authors:** Hussain-Abdulah Arjmand, Jesper Hohagen, Bryan Paton, Nikki S. Rickard

**Affiliations:** ^1^School of Psychological Sciences, Monash University, Melbourne, VIC, Australia; ^2^Institute for Systematic Musicology, University of Hamburg, Hamburg, Germany; ^3^Monash Biomedical Imaging, Monash University, University of Newcastle, Newcastle, NSW, Australia; ^4^Centre for Positive Psychology, Graduate School of Education, University of Melbourne, Melbourne, VIC, Australia

**Keywords:** frontal asymmetry, subjective emotions, pleasurable music, musicology, positive and negative affect

## Abstract

Recent studies have demonstrated increased activity in brain regions associated with emotion and reward when listening to pleasurable music. Unexpected change in musical features intensity and tempo – and thereby enhanced tension and anticipation – is proposed to be one of the primary mechanisms by which music induces a strong emotional response in listeners. Whether such musical features coincide with central measures of emotional response has not, however, been extensively examined. In this study, subjective and physiological measures of experienced emotion were obtained continuously from 18 participants (12 females, 6 males; 18–38 years) who listened to four stimuli—pleasant music, unpleasant music (dissonant manipulations of their own music), neutral music, and no music, in a counter-balanced order. Each stimulus was presented twice: electroencephalograph (EEG) data were collected during the first, while participants continuously subjectively rated the stimuli during the second presentation. Frontal asymmetry (FA) indices from frontal and temporal sites were calculated, and peak periods of bias toward the left (indicating a shift toward positive affect) were identified across the sample. The music pieces were also examined to define the temporal onset of key musical features. Subjective reports of emotional experience averaged across the condition confirmed participants rated their music selection as very positive, the scrambled music as negative, and the neutral music and silence as neither positive nor negative. Significant effects in FA were observed in the frontal electrode pair FC3–FC4, and the greatest increase in left bias from baseline was observed in response to pleasurable music. These results are consistent with findings from previous research. Peak FA responses at this site were also found to co-occur with key musical events relating to change, for instance, the introduction of a new motif, or an instrument change, or a change in low level acoustic factors such as pitch, dynamics or texture. These findings provide empirical support for the proposal that change in basic musical features is a fundamental trigger of emotional responses in listeners.

## Introduction

One of the most intriguing debates in music psychology research is whether the emotions people report when listening to music are ‘real.’ Various authorities have argued that music is one of the most powerful means of inducing emotions, from Tolstoy’s mantra that “music is the shorthand of emotion,” to the deeply researched and influential reference texts of Leonard Meyer (“Emotion and meaning in music”; Meyer, 1956) and Juslin and Sloboda (“The Handbook of music and emotion”; Juslin and Sloboda, 2010). Emotions evolved as a response to events in the environment which are potentially significant for the organism’s survival. Key features of these ‘utilitarian’ emotions include goal relevance, action readiness and multicomponentiality (Frijda and Scherer, 2009). Emotions are therefore triggered by events that are appraised as relevant to one’s survival, and help prepare us to respond, for instance via fight or flight. In addition to the cognitive appraisal, emotions are also widely acknowledged to be multidimensional, yielding changes in subjective feeling, physiological arousal, and behavioral response (Scherer, 2009). The absence of clear goal implications of music listening, or any need to become ‘action ready,’ however, challenges the claim that music-induced emotions are real (Kivy, 1990; Konecni, 2013).

A growing body of ‘emotivist’ music psychology research has nonetheless demonstrated that music does elicit a response in multiple components, as observed with non-aesthetic (or ‘utilitarian’) emotions. The generation of an emotion in subcortical regions of the brain (such as the amygdala) lead to hypothalamic and autonomic nervous system activation and release of arousal hormones, such as noradrenaline and cortisol. Sympathetic nervous system changes associated with physiological arousal, such as increased heart rate and reduced skin conductance, are most commonly measured as peripheral indices of emotion. A large body of work now illustrates, under a range of conditions and with a variety of music genres, that emotionally exciting or powerful music impacts on these autonomic measures of emotion (see Bartlett, 1996; Panksepp and Bernatzky, 2002; Hodges, 2010; Rickard, 2012 for reviews). For example, Krumhansl (1997) recorded physiological (heart rate, blood pressure, transit time and amplitude, respiration, skin conductance, and skin temperature) and subjective measures of emotion in real time while participants listened to music. The observed changes in these measures differed according to the emotion category of the music, and was similar (although not identical) to that observed for non-musical stimuli. Rickard (2004) also observed coherent subjective and physiological (chills and skin conductance) responses to music selected by participants as emotionally powerful, which was interpreted as support for the emotivist perspective on music-induced emotions.

It appears then that the evidence supporting music evoked emotions being ‘real’ is substantive, despite no obvious goal implications, or need for action, of this primarily aesthetic stimulus. Scherer and Coutinho (2013) have argued that music may induce a particular ‘kind’ of emotion – aesthetic emotions – that are triggered by novelty and complexity, rather than direct relevance to one’s survival. Novelty and complexity are nonetheless features of goal relevant stimuli, even though in the case of music, there is no significance to the listener’s survival. In the same way that secondary reinforcers appropriate the physiological systems of primary reinforcers via association, it is possible then that music may also hijack the emotion system by sharing some key features of goal relevant stimuli.

Multiple mechanisms have been proposed to explain how music is capable of inducing emotions (e.g., Juslin et al., 2010; Scherer and Coutinho, 2013). Common to most theories is an almost primal response elicited by psychoacoustic features of music (but also shared by other auditory stimuli). Juslin et al. (2010) describe how the ‘brain stem reflex’ (from their ‘BRECVEMA’ theory) is activated by changes in basic acoustic events – such as sudden loudness or fast rhythms – by tapping into an evolutionarily ancient survival system. This is because these acoustic events are associated with events that do in fact signal relevance for survival for real events (such as a nearby loud noise, or a rapidly approaching predator). Any unexpected *change* in acoustic feature, whether it be in pitch, timbre, loudness, or tempo, in music could therefore fundamentally be worthy of special attention, and therefore trigger an arousal response (Gabrielsson and Lindstrom, 2010; Juslin et al., 2010). Huron (2006) has elaborated on how music exploits this response by using extended anticipation and violation of expectations to intensify an emotional response. Higher level music events – such as motifs, or instrumental changes – may therefore also induce emotions via expectancy. In seminal work in this field, Sloboda (1991) asked participants to identify music passages which evoked strong, physical emotional responses in them, such as tears or chills. The most frequent musical events coded within these passages were new or unexpected harmonies, or appoggiaturas (which delay an expected principal note), supporting the proposal that unexpected musical events, or substantial changes in music features, were associated with physiological responses. Interestingly, a survey by Scherer et al. (2002) rated musical structure and acoustic features as more important in determining emotional reactions than the listener’s mood, affective involvement, personality or contextual factors. Importantly, because music events can elicit emotions via both expectation of an upcoming event and experience of that event, physiological markers of peak emotional responses may occur prior to, during or after a music event.

This proposal has received some empirical support via research demonstrating physiological peak responses to psychoacoustic ‘events’ in music (see **Table 1**). On the whole, changes in physiological arousal – primarily, chills, heart rate or skin conductance changes – coincided with sudden *changes* in acoustic features (such as changes in volume or tempo), or novel musical events (such as entry of new voices, or harmonic changes).

**Table 1:** Music features identified in the literature to be associated with various physiological markers of emotion.

Study	Physiological markers of emotion	Associated musical feature
Egermann et al. (2013)	Subjective and physiological arousal (skin conductance and heart rate)	Passages which violated expectations generated by a computational model which analyzed pitch features
Gomez and Danuser (2007)	Faster and higher minute ventilation, skin conductance, and heart rate	Tempo, accentuation, and rhythmic articulation Fast, accentuated, and staccato
Grewe et al. (2007a)	Chills	Entry of voice and changes in volume
Grewe et al. (2007b)	Skin conductance and facial muscle activity	The first entrance of a solo voice or choir and the beginning of new sections
Guhn et al. (2007)	Chills coinciding with distinct patterns of skin conductance increases	Passages which evoked chills had a number of similar characteristics: (1) they were from slow movements (adagio or larghetto), (2) they were characterized by alternation, or contrast, of the solo instrument and the orchestra, (3) a sudden or gradual volume increase from soft to loud, and (4) chill passages were characterized by an expansion in its frequency range in the high or low register, (5) all chill passages were characterized by harmonic peculiar progressions that potentially elicited ambiguity in the listener; that is, it deviated from what was expected based on the previous section
Coutinho and Cangelosi (2011)	Skin conductance and heart rate	Six low level music structural parameters: loudness, pitch level, pitch contour, tempo, texture, and sharpness
Steinbeis et al. (2006)	Electrodermal activity	Increases in harmonic unexpectedness

Supporting evidence for the similarity between music-evoked emotions and ‘real’ emotions has also emerged from research using central measures of emotional response. Importantly, brain regions associated with emotion and reward have been shown to also respond to emotionally powerful music. For instance, Blood and Zatorre (2001) found that pleasant music activated the dorsal amygdala (which connects to the ‘positive emotion’ network comprising the ventral striatum and orbitofrontal cortex), while reducing activity in central regions of the amygdala (which appear to be associated with unpleasant or aversive stimuli). Listening to pleasant music was also found to release dopamine in the striatum (Salimpoor et al., 2011, 2013). Further, the release was higher in the dorsal striatum during the anticipation of the peak emotional period of the music, but higher in the ventral striatum during the actual peak experience of the music. This is entirely consistent with the differentiated pattern of dopamine release during craving and consummation of other rewarding stimuli, e.g., amphetamines. Only one group to date has, however, attempted to identify musical features associated with central measures of emotional response. Koelsch et al. (2008a) performed a functional MRI study with musicians and non-musicians. While musicians tended to perceive syntactically irregular music events (single irregular chords) as slightly more pleasant than non-musicians, these generally perceived unpleasant events induced increased blood oxygen levels in the emotion-related brain region, the amygdala. Unexpected chords were also found to elicit specific event related potentials (ERAN and N5) as well as changes in skin conductance (Koelsch et al., 2008b). Specific music events associated with *pleasurable* emotions have not yet been examined using central measures of emotion.

Davidson and Irwin (1999), Davidson (2000, 2004), and Davidson et al. (2000), have demonstrated that a left bias in frontal cortical activity is associated with positive affect. Broadly, a left bias frontal asymmetry (FA) in the alpha band (8–13 Hz) has been associated with a positive affective style, higher levels of wellbeing and effective emotion regulation (Tomarken et al., 1992; Jackson et al., 2000). Interventions have been demonstrated to shift frontal electroencephalograph (EEG) activity to the left. An 8-week meditation training program significantly increased left sided FA when compared to wait list controls (Davidson et al., 2003). Blood et al. (1999) observed that left frontal brain areas were more likely to be activated by pleasant music than by unpleasant music. The amygdala appears to demonstrate valence-specific lateralization with pleasant music increasing responses in the left amygdala and unpleasant music increasing responses in the right amygdala (Brattico, 2015; Bogert et al., 2016). Positively valenced music has also been found to elicit greater frontal EEG activity in the left hemisphere, while negatively valenced music elicits greater frontal activity in the right hemisphere (Schmidt and Trainor, 2001; Altenmüller et al., 2002; Flores-Gutierrez et al., 2007). The pattern of data in these studies suggests that this frontal lateralization is mediated by the emotions *induced* by the music, rather than just the emotional valence they *perceive* in the music. Hausmann et al. (2013) provided support for this conclusion via mood induction through a musical procedure using happy or sad music, which reduced the right lateralization bias typically observed for emotional faces and visual tasks, and increased the left lateralization bias typically observed for language tasks. A right FA pattern associated with depression was found to be shifted by a music intervention (listening to 15 min of ‘uplifting’ popular music previously selected by another group of adolescents) in a group of adolescents (Jones and Field, 1999). This measure therefore provides a useful objective marker of emotional response to further identify whether specific music events are associated with physiological measures of emotion.

The aim in this study was to examine whether: (1) music perceived as ‘emotionally powerful’ and pleasant by listeners also elicited a response in a central marker of emotional response (frontal alpha asymmetry), as found in previous research; and (2) peaks in frontal alpha asymmetry were associated with changes in key musical or psychoacoustic events associated with emotion. To optimize the likelihood that emotions were induced (that is, felt rather than just perceived), participants listened to their own selections of highly pleasurable music. Two validation hypotheses were proposed to confirm the methodology was consistent with previous research. It was hypothesized that: (1) emotionally powerful and pleasant music selected by participants would be rated as more positive than silence, neutral music or a dissonant (unpleasant) version of their music; and (2) emotionally powerful pleasant music would elicit greater shifts in frontal alpha asymmetry than control auditory stimuli or silence. The primary novel hypothesis was that peak alpha periods would coincide with changes in basic psychoacoustic features, reflecting unexpected or anticipatory musical events. Since music-induced emotions can occur both before and after key music events, FA peaks were considered associated with music events if the music event occurred within 5 s before to 5 s after the FA event. Music background and affective style were also taken into account as potential confounds.

## Materials and Methods

### Participants

The sample for this study consisted of 18 participants (6 males, 12 females) recruited from tertiary institutions located in Melbourne, Australia. Participants’ ages ranged between 18 and 38 years (*M* = 22.22, *SD* = 5.00). Participants were excluded if they were younger than 17 years of age, had an uncorrected hearing loss, were taking medication that may impact on mood or concentration, were left-handed, or had a history of severe head injuries or seizure-related disorder. Despite clearly stated exclusion criteria, two left handed participants attended the lab, although as the pattern of their hemispheric activity did not appear to differ to right-handed participants, their data were retained. Informed consent was obtained through an online questionnaire that participants completed prior to the laboratory session.

### Materials

#### Online Survey

The online survey consisted of questions pertaining to demographic information (gender, age, a left-handedness question, education, employment status and income), music background (MUSE questionnaire; Chin and Rickard, 2012) and affective style (PANAS; Watson and Tellegen, 1988). The survey also provided an anonymous code to allow matching with laboratory data, instructions for attending the laboratory and music choices, and explanatory information about the study and a consent form.

#### Peak Frontal Asymmetry in Alpha EEG Frequency Band

The physiological index of emotion was measured using electroencephalography (EEG). EEG data were recorded using a 64-electrode silver-silver chloride (Ag-AgCl) EEG elastic Quik-cap (Compumedics) in accordance with the international 10–20 system. Data are, however, analyzed and reported from midfrontal sites (F3/F4 and FC3/FC4) only, as hemispheric asymmetry associated with positive and negative affect has been observed primarily in frontal cortex (Davidson et al., 1990; Tomarken et al., 1992; Dennis and Solomon, 2010). Further spatial exploration of data for structural mapping purposes was beyond of the scope of this paper. In addition, analyses were performed for the P3–P4 sites as a negative control (Schmidt and Trainor, 2001; Dennis and Solomon, 2010). All channels were referenced to the mastoid electrodes (M1–M2). The ground electrode was situated between FPZ and FZ and impedances were kept below 10 kOhms. Data were collected and analyzed offline using the Compumedics Neuroscan 4.5 software.

#### Subjective Emotional Response

The subjective feeling component of emotion was measured using ‘EmuJoy’ software (Nagel et al., 2007). This software allows participants to indicate how they feel in real time as they listen to the stimulus by moving the cursor along the screen. The Emujoy program utilizes the circumplex model of affect (Russell, 1980) where emotion is measured in a two dimensional affective space, with axes of arousal and valence. Previous studies have shown that valence and arousal account for a large portion of the variation observed in the emotional labeling of musical (e.g., Thayer, 1986), as well as linguistic (Russell, 1980) and picture-oriented (Bradley and Lang, 1994) experimental stimuli. The sampling rate was 20 Hz (one sample every 50 ms), which is consistent with recommendations for continuous monitoring of subjective ratings of emotion (Schubert, 2010). Consistent with Nagel et al. (2007), the visual scale was quantified as an interval scale from -10 to +10.

#### Music Stimuli

Four music stimuli—practice, pleasant, unpleasant, and neutral—were presented throughout the experiment. Each stimulus lasted between 3 and 5 min in duration. The practice stimulus was presented to familiarize participants with the Emujoy program and to acclimatize participants to the sound and the onset and offset of the music stimulus (fading in at the start and fading out at the end). The song was selected on the basis that it was likely to be familiar to participants, positive in affective valence, and containing segments of both arousing and calming music—The Lion King musical theme song, “*The circle of life.*”

The pleasant music stimulus was participant-selected. This option was preferred over experimenter-selected music as participant-selected music was considered more likely to induce robust emotions (Thaut and Davis, 1993; Panksepp, 1995; Blood and Zatorre, 2001; Rickard, 2004). Participants were instructed to select a music piece that made them, “experience positive emotions (happy, joyful, excited, etc.) – like those songs you absolutely love or make you get goose bumps.” This song selection also had to be one that would be considered a happy song by the general public. That is, it could not be sad music that participants enjoyed. While previous research has used both positively and negatively valenced music to elicit strong experiences with music, in the current study, we limited the music choices to those that expressed positive emotions. This decision was made to reduce variability in EEG responses arising from perception of negative emotions and experience of positive emotions, as EEG can be sensitive to differences in both perception and experience of emotional valence. The music also had to be alyrical^1^—music with unintelligible words, for example in another language or skat singing, were permitted—as language processing might conceivably elicit different patterns of hemisphere activation solely as a function of the processing of vocabulary included in the song. [It should be noted that there are numerous mechanisms by which a piece of music might induce an emotion (see Juslin and Vastfjall, 2008), including associations with autobiographical events, visual imagery and brain stem reflexes. Differentiating between these various causes of emotion was, however, beyond the scope of the current study.]

The unpleasant music stimulus was intended to induce negative emotions. This was a dissonant piece produced by manipulating the participant’s pleasant music stimulus and was achieved using Sony Sound Forge© 8 software. This stimulus consisted of three versions of the song played simultaneously— one shifted a tritone down, one pitch shifted a whole tone up, and one played in reverse (adapted from Koelsch et al., 2006). The neutral condition was an operatic track, La Traviata, chosen based upon its neutrality observed in previous research (Mitterschiffthaler et al., 2007).

The presentation of music stimuli was controlled by the experimenter via the EmuJoy program. The music volume was set to a comfortable listening level, and participants listened to all stimuli via bud earphones (to avoid interference with the EEG cap).

### Procedure

Prior to attending the laboratory session, participants completed the anonymously coded online survey. Within 2 weeks, participants attended the EEG laboratory at the Monash Biomedical Imaging Centre. Participants were tested individually during a 3 h session. An identification code was requested in order to match questionnaire data with laboratory session data.

**FIGURE 1:**
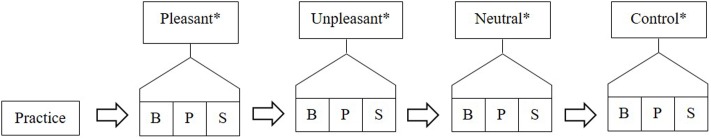
Example of testing structure with conditions ordered; pleasant, unpleasant, neutral, and control. B, baseline; P, physiological recording; and S, subjective rating. ^∗^These stimuli were presented to participants in a counter balanced order.

Participants were seated in a comfortable chair and were prepared for fitting of the EEG cap. The participant’s forehead was cleaned using medical grade alcohol swabs and exfoliated using NuPrep exfoliant gel. Participants were fitted with the EEG cap according to the standardized international 10/20 system (Jasper, 1958). Blinks and vertical/horizontal movements were recorded by attaching loose electrodes from the cap above and below the left eye, as well as laterally on the outer canthi of each eye. The structure of the testing was explained to participants and was as follows (see **Figure 1**):

The testing comprised four within-subjects conditions: pleasant, unpleasant, neutral, and control. Differing only in the type of auditory stimulus presented, each condition consisted of:

(a) Baseline recording (B)—no stimulus was presented during the baseline recordings. These lasted 3 min in duration and participants were asked to close their eyes and relax.
(b) Physiological recording (P)—the stimulus (depending on what condition) was played and participants were asked to have their eyes closed and to just experience the sounds.
(c) Subjective rating (S)—the stimulus was repeated, however, this time participants were asked to indicate, with eyes open, how they felt as they listened to the same music on the computer screen using the cursor and the EmuJoy software.

At every step of each condition, participants were guided by the experimenter (e.g., “I’m going to present a stimulus to you now, it may be music, something that sounds like music, or it could be nothing at all. All I would like you to do is to close your eyes and just experience the sounds”). Before the official testing began, the participant was asked to practice using the EmuJoy program in response to the practice stimulus. Participants were asked about their level of comfort and understanding with regards to using the EmuJoy software; experimentation did not begin until participants felt comfortable and understood the use of EmuJoy. Participants were reminded of the distinction between rating emotions *felt* vs. emotions *perceived* in the music; the former was encouraged throughout relevant sections of the experiment. After this, the experimental procedure began with each condition being presented to participants in a counterbalanced fashion. All procedures in this study were approved by the Monash University Human Research Ethics Committee.

### EEG Data Analysis for Frontal Asymmetry

Electroencephalograph data from each participant was visually inspected for artifacts (eye movements and muscle artifacts were manually removed prior to any analyses). EEG data were also digitally filtered with a low-pass zero phase-shift filter set to 30 Hz and 96 dB/oct. All data were re-referenced to mastoid processes. The sampling rate was 1250 Hz and eye movements were controlled for with automatic artifact rejection of >50 μV in reference to VEO. Data were baseline corrected to 100 ms pre-stimulus period. EEG data were aggregated for all artifact-free periods within a condition to form a set of data for the positive music, negative music, neutral, and the control.

**FIGURE 2:**
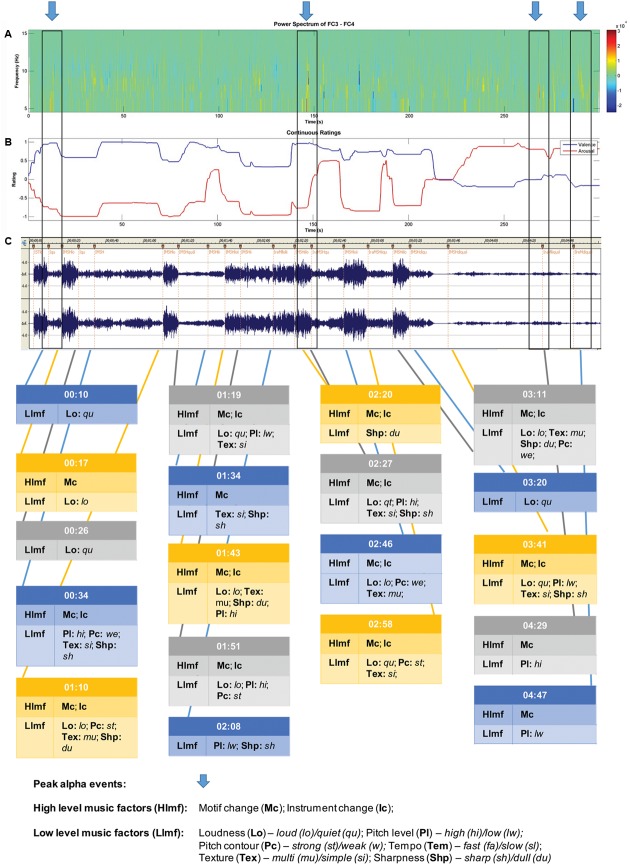
Sample data for participant 4 – music selection: The Four Seasons: Spring; Antonio Vivaldi. Recording: Karoly Botvay, Budapest Strings, Cobra Entertainment). **(A)** EEG alpha band spectrogram; **(B)** subjective valence and arousal ratings; and **(C)** music feature analysis.

Chunks of 1024 ms were extracted for analyses using a Cosine window. A Fast Fourier Transform (FFT) was applied to each chunk of EEG permitting the computation of the amount of power at different frequencies. Power values from all chunks within an epoch were averaged (see Dumermuth and Molinari, 1987). The dependent measure that was extracted from this analysis was power density (μV^2^/Hz) in the alpha band (8–13 Hz). The data were log transformed to normalize their distribution because power values are positively skewed (Davidson, 1988). Power in the alpha band is inversely related to activation (e.g., Lindsley and Wicke, 1974) and has been the measure most consistently obtained in studies of EEG asymmetry (Davidson, 1988). Cortical asymmetry [ln(right)–ln(left)] was computed for the alpha band. This FA score provides a simple unidimensional scale representing relative activity of the right and left hemispheres for an electrode pair (e.g., F3 [left]/F4 [right]). FA scores of 0 indicate no asymmetry, while scores greater than 0 putatively are indicative of greater left frontal activity (positive affective response) and scores below 0 are indicative of greater right frontal activity (negative affective response), assuming that alpha is inversely related to activity (Allen et al., 2004). Peak FA periods at the FC3/FC4 site were also identified across each participant’s pleasant music piece for purposes of music event analysis. FA (difference between left and right power densities) values were ranked from highest (most asymmetric, left biased) to lowest using spectrograms (see **Figure 2** for an example). Due to considerable inter-individual variability in asymmetry ranges, descriptive ranking was used as a selection criterion instead of an absolute threshold or statistical difference criterion. The ranked FA differences were inspected and those that were clearly separated from the others (on average six peaks were clearly more asymmetric than the rest of the record) were selected for each individual as their greatest moments of FA. This process was performed by two raters (authors H-AA and NR), with 100% interrater reliability, so no further analysis was performed/considered necessary required to rank the FA peaks.

### Music Event Data Coding

A subjective method of annotating each pleasant music piece with temporal onsets and types of all notable changes in musical features was utilized in this study. Coding was performed by a music performer and producer with postgraduate qualifications in systematic musicology. A decision was made to use subjective coding as it has been successfully used previously to identify significant changes in a broad range of music features associated with emotional induction by music (Sloboda, 1991). This method was framed within a hierarchical category model which contained both low-level and high-level factors of important changes. First, each participant’s music piece was described by annotating the audiogram, noting the types of music changes at respective times. Secondly, the low-level factor model utilized by Coutinho and Cangelosi (2011) was applied to assign the identified music features deductively to changes within six low-level factors: loudness, pitch level, pitch contour, tempo, texture, and sharpness. Each low-level factor change was coded as a change toward one of the two anchors of the feature. For example, if a modification was marked in terms of loudness with ‘loud,’ it described an increase in loudness of the current part compared to the part before (see **Table 2**).

**Table 2:** Operational definitions of high and low level musical features investigated in the current study.

Music feature	Operational definition
**High level factors**	
Motif changes	A theme/movement/motif of the leading melody changes from one part to another (e.g., from one motif to another/from first to second movement/from verse to bridge to chorus)
Instrumentation changes	the number and/or type of instruments played in one part is different compared to the part before
**Low level factors**	
Loudness	Is **loud**, if the general sound of one part is louder compared to the part before
Pitch level	Is **high**, if the pitch level of the leading melody is higher compared to the part before
Pitch contour	Is **strong**, if there are more pitch changes in the leading melody of one part compared to the number of changes in the part before
Tempo	Is **fast**, if the perceived tempo of one part is faster compared to the part before
Texture	Is **multi**, if there are more musical moments in which a high number of tones are playing simultaneously in one part compared to the part before
Sharpness	Is **sharp**, if there are more single differences in loudness and timbre from the different instruments or tones in one part compared to the part before

Due to the high variability of the analyzed musical pieces from a musicological perspective – including the genre, which ranged from classical and jazz to pop and electronica – every song had a different frequency of changes in terms of these six factors. Hence, we applied a third step of categorization which led to a more abstract layer of changes in musical features that included two higher-level factors: motif changes and instrument changes. A time point in the music is marked with ‘motif change’ if the theme, movement or motif of the leading melody change from one part to the next one. The factor ‘instrument change’ can be defined as an increase or decrease of the number of playing instruments or as a change of instruments used within the current part.

## Results

Data were scored and entered into PASW Statistics 18 for analyses. No missing data or outliers were observed in the survey data. Bivariate correlations were run between potential confounding variables – Positive affect negative affect schedule (PANAS), and the Music use questionnaire (MUSE) – and FA to determine if they were potential confounds, but no correlations were observed.

A sample of data obtained for each participant is shown in **Figure 2**. For this participant, five peak alpha periods were identified (shown in blue arrows at top). Changes in subjective valence and arousal across the piece are shown in the second panel, and then the musicological analysis in the final section of the figure.

### Subjective Ratings of Emotion – Averaged Emotional Responses

**FIGURE 3:**
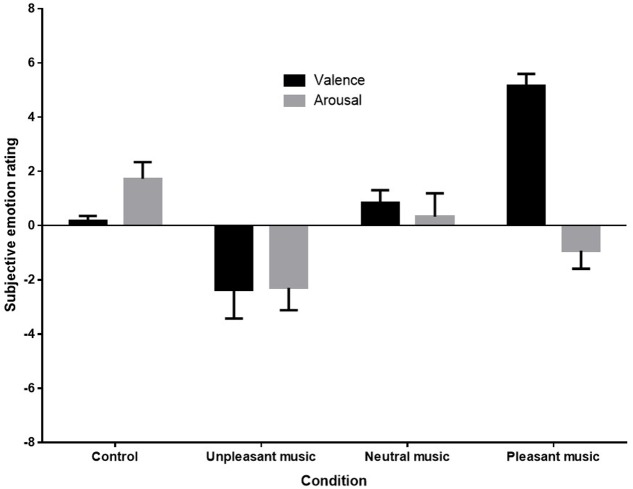
Mean subjective emotion ratings (valence and arousal) for control (silence), unpleasant (dissonant), neutral, and pleasant (self-selected) music conditions.

A one-way analysis of variance (ANOVA) was conducted to compare mean subjective ratings of emotional valence. Kolmogorov–Smirnov tests of normality indicated that distributions were normal for each condition except the subjective ratings of the control condition *D*(9) = 0.35, *p* < 0.001. Nonetheless, as ANOVAs are robust to violations of normality when group sizes are equal (Howell, 2002), parametric tests were retained. No missing data or outliers were observed in the subjective rating data. **Figure 3** below shows the mean ratings of each condition.

**Figure 3** shows that both the direction and magnitude of subjective emotional valence differed across conditions, with the pleasant condition rated very positively, the unpleasant condition rated negatively, and the control and neutral conditions rated as neutral. Arousal ratings appeared to be reduced in response to unpleasant and pleasant music. (Anecdotal reports from participants indicated that in addition to being very familiar with their own music, participants recognized the unpleasant piece as a dissonant manipulation of their own music selection, and were therefore familiar with it also. Several participants noted that this made the piece even more unpleasant to listen to for them.)

Sphericity was met for the arousal ratings, but not for valence ratings, so a Greenhouse-Geisser correction was made for analyses on valence ratings. A one-way repeated measures ANOVA revealed a significant effect of stimulus condition on valence ratings, *F*(1.6,27.07) = 23.442, *p* < 0.001, $\eta_{p}^{2}$ = 0.58. *Post hoc* contrasts revealed that the mean subjective valence rating for the unpleasant music was significantly lower than for the control *F*(1,17) = 5.59, *p* = 0.030, $\eta_{p}^{2}$ = 0.25, and the mean subjective valence rating for the pleasant music was significantly higher than for the control condition, *F*(1,17) = 112.42, *p* < 0.001, $\eta_{p}^{2}$ = 0.87. The one-way repeated measures ANOVA for arousal ratings also showed a significant effect for stimulus condition, *F*(3,51) = 5.20, *p* = 0.003, $\eta_{p}^{2}$ = 0.23. *Post hoc* contrasts revealed that arousal ratings were significant reduced by both unpleasant, *F*(1,17) = 10.11, *p* = 0.005, $\eta_{p}^{2}$ = 0.37, and pleasant music, *F*(1,17) = 6.88, *p* = 0.018, $\eta_{p}^{2}$ = 0.29, when compared with ratings for the control.

### Aim 1: Can Emotionally Pleasant Music Be Detected by a Central Marker of Emotion (FA)?

Two-way repeated measures ANOVAs were conducted on the FA scores (averaged across baseline period, and averaged across condition) for each of the two frontal electrode pairs, and the control parietal site pair. The within-subjects factor included the music condition (positive, negative, neutral, and control) and time (baseline and stimulus). Despite the robustness of ANOVA to assumptions, caution should be taken in interpreting results as both the normality and sphericity assumptions were violated across each electrode pair. Where sphericity was violated, a Greenhouse–Geisser correction was applied. Asymmetry scores above two were considered likely a result of noisy or damaged electrodes (62 points out of 864) and were omitted as missing data which were excluded pairwise. Two outliers were identified in the data and were replaced with a score ±3.29 standard deviations from the mean.

**FIGURE 4:**
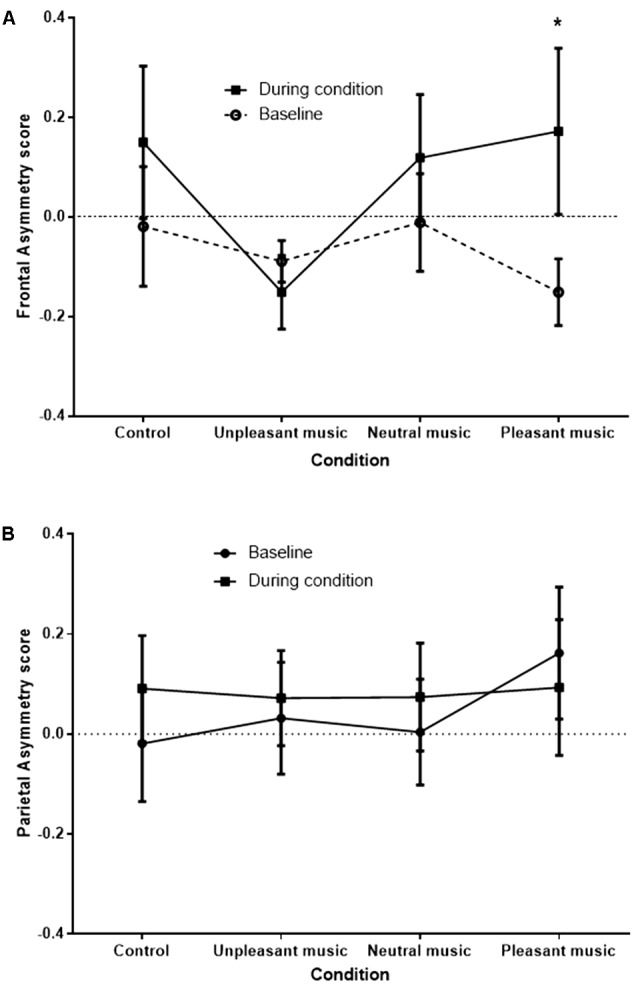
FC3/FC4 **(A)** and P3/P4 **(B)** (control) asymmetry score at baseline and during condition, for each condition. Asymmetry scores of 0 indicate no asymmetry. Scores >0 indicate left bias asymmetry (and positive affect), while scores <0 indicate right bias asymmetry (and negative affect). ^∗^*p* < 0.05.

A signification time by condition interaction effect was observed at the FC3/FC4 site, *F*(2.09,27.17) = 3.45, *p* = 0.045, $\eta_{p}^{2}$ = 0.210, and a significant condition main effect was observed at the F3/F4 site, *F*(2.58,21.59) = 3.22, *p* = 0.039, $\eta_{p}^{2}$ = 0.168. No significant effects were observed at the P3/P4 site [time by condition effect, *F*(1.98,23.76) = 2.27, *p* = 0.126]. The significant interaction at FC3/FC4 is shown in **Figure 4**.

The greatest difference between baseline and during condition FA scores was for the pleasant music, representative of a positive shift in asymmetry from the right hemisphere to the left when comparing the baseline period to the stimulus period. Planned simple contrasts revealed that when compared with the unpleasant music condition, only the pleasant music condition showed a significant positive shift in FA score, *F*(1,13) = 6.27, *p* = 0.026. Positive shifts in FA were also apparent for control and neutral music conditions, although not significantly greater than for the unpleasant music condition [*F*(1,13) = 2.60, *p* = 0.131, and *F*(1,13) = 3.28, *p* = 0.093], respectively.

### Aim 2: Are Peak FA Periods Associated with Particular Musical Events?

Peak periods of FA were identified for each participant, and the sum varied between 2 and 9 (*M* = 6.5, *SD* = 2.0). The music event description was then examined for presence or absence of coded musical events within a 10 s time window of (5 s before to 5 s after) the peak FA time-points. Across all participants, 106 peak alpha periods were identified, 78 of which (74%) were associated with particular music events. The type of music event coinciding with peak alpha periods is shown in **Table 3**. A two-step cluster analysis was also performed to explore natural groupings of peak alpha asymmetry events that coincided with distinct *combinations* (2 or more) of musical features. A musical feature was to be deemed a salient characteristic of a cluster if present in at least 70% of the peak alpha events within the same cluster.

**Table 3:** Frequency and percentages of musical features associated with a physiological marker of emotion (peak alpha FA). High level, low level, and clusters of music features are distinguished.

Music-structural	Frequency	Percentage
features	during peak	during peak
	alpha periods	alpha periods
**High level music factors**		
Motif change	51	65.4
Instrument change	38	48.7
**Low level music factors**		
Pitch (high)	35	44.9
Loudness (loud)	20	25.6
Texture (simple)	11	14.1
Pitch contour (strong)	18	23.1
Sharpness (dull)	15	19.2
Sharpness (sharp)	14	18.0
Pitch (low)	14	18.0
Loudness (soft)	14	18.0
Texture (multi)	19	24.3
Pitch contour (weak)	7	9.0
Tempo (slow)	2	2.6
Tempo (fast)	1	1.2
**Feature cluster 1 (14/78 alpha periods)**		
Motif change	10	71
Instrument change	14	100
Texture (multi)	12	86
Sharpness (dull)	13	93
**Feature cluster 2 (11 of 78 alpha periods)**		
Motif change	8	73
Loudness (high level factor)	11	100
Loudness (loud; low level factor)	11	100
**Feature cluster 3 (14 of 78 alpha periods)**		
Motif change	10	71
Instrument change	11	79
Softness (high level factor)	14	100
Loudness (soft, low level factor)	12	86

**Table 3** shows that, considered independently, the most frequent music features associated with peak alpha periods were primarily high level factors (changes in motif and instruments), with the addition of one low level factor (pitch). In exploring the data for clusters of peak alpha events associated with combinations of musical features, a four cluster solution was found to successfully group approximately half (53%) of the events into groups with identifiable patterns. This equated to 3 separate clusters characterized by distinct combinations of musical features, with the remaining half (47%) deemed unclassifiable as higher factor solutions provided no further differentiation.

## Discussion

In the current study, a central physiological marker (alpha FA) was used to investigate the emotional response of music selected by participants to be ‘emotionally powerful’ and pleasant. Musical features of these pieces were also examined to explore associations between key musical events and central physiological markers of emotional responding. The first aim of this study was to examine whether pleasant music elicited physiological reactions in this central marker of emotional responding. As hypothesized, pleasant musical stimuli elicited greater shifts in FA than did the control auditory stimulus, silence or an unpleasant dissonant version of each participant’s music. This finding confirmed previous research findings and demonstrated that the methodology was robust and appropriate for further investigation. The second aim was to examine associations between key musical features (affiliated with emotion), contained within participant-selected musical pieces, and peaks in FA. FA peaks were commonly associated with changes in both high and low level music features, including changes in motif, instrument, loudness and pitch, supporting the hypothesis that key events in music are marked by significant physiological changes in the listener. Further, specific combinations of individual musical features were identified that tended to predict FA peaks.

### Central Physiological Measures of Responding to Musical Stimuli

Participants’ subjective valence ratings of music were consistent with expectations; control and neutral music were both rated neutrally, while unpleasant music was rated negatively and pleasant music was rated very positively. These findings are consistent with previous research indicating that music is capable of eliciting strong *felt* positive affective reports (Panksepp, 1995; Rickard, 2004; Juslin et al., 2008; Zenter et al., 2008; Eerola and Vuoskoski, 2011). The current findings were also consistent with previous negative subjective ratings (unpleasantness) by participants listening to the dissonant manipulation of musical stimuli (Koelsch et al., 2006). It is not entirely clear why arousal ratings were reduced by both the unpleasant and pleasant music. The variety of pieces selected by participants means that both relaxing and stimulating pieces were present in these conditions, although overall, the findings suggest that listening to music (regardless of whether pleasant or unpleasant) was more calming than silence for this sample. In addition, as both pieces were likely to be familiar (as participants reported that they recognized the dissonant manipulations of their own piece), familiarity could have reduced the arousal response expected for unpleasant music.

As hypothesized, FA responses from the FC3/FC4 site were consistent with subjective valence ratings, with the largest shift to the left hemisphere observed for the pleasant music condition. While not statistically significant, the small shifts to the left hemisphere during both control and neutral music conditions, and the small shift to the right hemisphere during the unpleasant music condition, indicate the trends in FA were also consistent with subjective valence reports. These findings support previous research findings on the involvement of the left frontal lobe in positive emotional experiences, and the right frontal lobe in negative emotional experiences (Davidson et al., 1979, 1990; Fox and Davidson, 1986; Davidson and Fox, 1989; Tomarken et al., 1990). The demonstration of these effects in the FC3/FC4 site is consistent with previous findings (Davidson et al., 1990; Jackson et al., 2003; Travis and Arenander, 2006; Kline and Allen, 2008; Dennis and Solomon, 2010), although meaningful findings are also commonly obtained from data collected from the F3/F4 site (see Schmidt and Trainor, 2001; Thibodeau et al., 2006), which was not observed in the current study. The asymmetry findings also verify findings observed in response to positive and negative emotion induction by music (Schmidt and Trainor, 2001; Altenmüller et al., 2002; Flores-Gutierrez et al., 2007; Hausmann et al., 2013). Importantly, no significant FA effect was observed in the control P3/P4 sites, which is an area not implicated in emotional responding.

### Associations between Musical Features and Peak Periods of Frontal Asymmetry

#### Individual Musical Features

Several individual musical features coincided with peak FA events. Each of these musical features occurred in over 40% of the total peak alpha asymmetry events identified throughout the sample and appear to be closely related to *changes* in musical structure. These included changes in motif and instruments (high level factors), as well as pitch (low level factor). Such findings are in line with previous studies measuring non-central physiological measures of affective responding. For example, high level factor musical features such as instrument change, specifically changes and alternations between orchestra and solo piece instruments have been cited to coincide with chill responses (Grewe et al., 2007b; Guhn et al., 2007). Similarly, pitch events have been observed in previous research to coincide with various physiological measures of emotional responding including skin conductance and heart rate (Coutinho and Cangelosi, 2011; Egermann et al., 2013). In the current study, instances of *high* pitch were most closely associated with physiological reactions. These findings can be explained through Juslin and Sloboda’s (2010) description of the activation of a ‘brain stem reflex’ in response to changes in basic acoustic events. Changes in loudness and high pitch levels may trigger physiological reactions on account of being psychoacoustic features of music that are shared with more primitive auditory stimuli that signal relevance for survival to real events.

Changes in instruments and motif, however, may be less related to primitive auditory stimuli and stimulate physiological reactions differently. Motif changes have not been observed in previous studies yet appeared most frequently throughout the peak alpha asymmetry events identified in the sample. In music, motif has been described as “...the smallest structural unit possessing thematic identity” (White, 1976, p. 26–27) and exists as a salient and recurring characteristic musical fragment throughout a musical piece. Within the descriptive analysis of the current study, however, a *motif* can be understood in a much broader sense (see definitions in **Table 2**). Due to the broad musical diversity of the songs selected by participants, the term *motif change* emerged as most appropriate description to cover high level structural changes in all the different musical pieces (e.g., changes from one small unit to another in a classic piece and changes from a long repetitive pattern to a chorus in an electronic dance piece). Changes in such a fundamental musical feature, as well as changes in instrument, are likely to stimulate a sense of novelty and add complexity, and possibly unexpectedness (i.e., features of goal oriented stimuli), to a musical piece. This may therefore also recruit the same neural system which has evolved to yield an emotional response, which in this study, is manifest in the greater activation in the left frontal hemisphere compared to the right frontal hemisphere. Many of the other musical features identified, however, did not coincide frequently with peak FA events. While peripheral markers of emotion, such as skin conductance and heart rate changes, are likely to respond strongly to basic psychoacoustic events associated with arousal, it is likely that central markers such as FA are more sensitive to higher level musical events associated with positive affect. This may explain why motif changes were a particularly frequent event associated with FA peaks. Alternatively, some musical features may evoke emotional and physiological reactions only when present in conjunction with other musical features. It is recognized that an objective method of low level music feature identification would also be useful in future research to validate the current findings relating to low level psychoacoustic events. A limitation of the current study, however, was that the coding of both peak FA events and music events was subjective, which limits both replicability and objectivity. It is recommended future research utilize more objective coding techniques including statistical identification of peak FA events, and formal psychoacoustic analysis (such as achieved using software tools such as MIR Toolbox or PsySound). While an objective method of detecting FA events occurring within a specific time period after a music event is also appealing, the current methodology operationalized synchrony of FA and music events within a 10 s time window to include mechanisms of anticipation as well as experience of the event. Future research may be able to provide further distinction between these emotion induction mechanisms by applying different time windows to such analyses.

#### Feature Clusters of Musical Feature Combinations

Several clusters comprising combinations of musical features were identified in the current study. A number of musical events which on their own did not coincide with FA peaks did nonetheless appear in music event clusters that were associated with FA peaks. For example, feature cluster 1 consists of motif and instrument changes—both individually considered to coincide frequently with peak alpha asymmetry events—as well as texture (multi) and sharpness (dull). Changes in texture and sharpness, individually, were observed to occur in only 24.3 and 19.2% of the total peak alpha asymmetry events, respectively. After exploring the data for natural groupings of musical events that occurred during peak alpha asymmetry scores, however, texture and sharpness changes appeared to occur frequently in conjunction with motif changes and instrument changes. Within feature cluster 1, texture and sharpness occurred in 86 and 93% of the peak alpha asymmetry periods. This suggests that certain musical features, like texture and sharpness, may lead to stronger emotional responses in central markers of physiological functioning when presented concurrently with specific musical events as compared to instances where they are present in isolation.

An interesting related observation is the specificity with which these musical events can combine to form a cluster. While motif and instrument changes occurred often in conjunction with texture (multi) and sharpness (dull) during peak alpha asymmetry events, both also occurred distinctly in conjunction with dynamic changes in volume (high level factor) and softness (low level factor) in a separate feature cluster. While both the texture/sharpness and loudness change/softness combinations frequently occur with motif and instrument changes, they appear to do so in a mutually exclusive manner. This suggests a high level of complexity and specificity with which musical features may complement one another to stimulate physiological reactions during musical pieces.

The current findings extend previous research which has demonstrated that emotionally powerful music elicits changes in physiological, as well as subjective, measures of emotion. This study provides further empirical support for the emotivist theory of music and emotion which proposes that if emotional responses to music are ‘real,’ then they should be observable in physiological indices of emotion (Krumhansl, 1997; Rickard, 2004). The pattern of FA observed in this study is consistent with that observed in previous research in response to positive and negative music (Blood et al., 1999; Schmidt and Trainor, 2001), and non-musical stimuli (Fox, 1991; Davidson, 1993, 2000). However, the current study utilized music which expressed and induced positive emotions only, whereas previous research has also included powerful emotions induced by music expressing negative emotions. It would be of interest to replicate the current study with a broader range of powerful music to determine whether FA is indeed a marker of emotional experience, or a mixture of emotion perception and experience.

The findings also extend those obtained in studies which have examined musical features associated with strong emotional responses. Consistent with the broad consensus in this research, strong emotional responses often coincide with music events that signal change, novelty or violated expectations (Sloboda, 1991; Huron, 2006; Steinbeis et al., 2006; Egermann et al., 2013). In particular, FA peaks were found to be associated in the current sample’s music selections with motif changes, instrument changes, dynamic changes in volume, and pitch, or specific clusters of music events. Importantly, however, these conclusions are limited by the modest sample size, and consequently by the music pieces selected. Further research utilizing a different set of music pieces may identify a quite distinct pattern of music features associated with FA peaks. In sum, these findings provide empirical support for anticipation/expectation as a fundamental mechanism underlying music’s capacity to evoke strong emotional responses in listeners.

## Ethics Statement

This study was carried out in accordance with the recommendations of the National Statement on Ethical Conduct in Human Research, National Health and Medical Research Council, with written informed consent from all subjects. All subjects gave written informed consent in accordance with the Declaration of Helsinki. The protocol was approved by the Monash University Standing Committee for Ethical Research on Humans.

## Author Contributions

H-AA conducted the experiments, contributed to the design and methods of the study, analysis of data and preparation of all sections of the manuscript. NR contributed to the design and methods of the study, analysis of data and preparation of all sections the manuscript, and provided oversight of this study. JH conducted the musicological analyses of the music selections, and contributed to the methods and results sections of the manuscript. BP performed the analyses of the EEG recordings and contributed to the methods and results sections of the manuscript.

## Conflict of Interest Statement

The authors declare that the research was conducted in the absence of any commercial or financial relationships that could be construed as a potential conflict of interest.

## Footnotes

1 One participant only chose music with lyrical content; the experimenter confirmed with this participant that the language (Italian) was unknown to them.
